# Dielectric Behavior of Thin Polymerized Composite Layers Fabricated by Inkjet-Printing

**DOI:** 10.3390/nano13030441

**Published:** 2023-01-21

**Authors:** Timo Reinheimer, Tim P. Mach, Kevin Häuser, Michael J. Hoffmann, Joachim R. Binder

**Affiliations:** Institute for Applied Materials, Karlsruhe Institute of Technology, 76344 Eggenstein-Leopoldshafen, Germany

**Keywords:** inkjet printing, printed capacitors, ceramic/polymer composites, dielectric behavior

## Abstract

A detailed study of the dielectric behavior of printed capacitors is given, in which the dielectric consists of a thin (<1 µm) ceramic/polymer composite layer with high permittivities of ε_r_ 20–69. The used ink contains surface-modified Ba_0.6_Sr_0.4_TiO_3_ (BST), a polymeric crosslinking agent and a thermal initiator, which allows the immediate polymerization of the ink during printing, leading to homogenous layers. To validate the results of the calculated permittivities, different layer thicknesses of the dielectric are printed and the capacitances, as well as the loss factors, are measured. Afterwards, the exact layer thicknesses are determined with cross sectional SEM images of ion-etched samples. Then, the permittivities are calculated with the known effective area of the capacitors. Furthermore, the ink composition is varied to obtain different ceramic/polymer ratios and thus different permittivities. The packing density of all composites is analyzed via SEM to show possible pores and validate the target ratio, respectively. The correlation between the chosen ratio and the measured permittivity is discussed using models from the literature. In addition, the leakage current of some capacitors is measured and discussed. For that, the dielectric was printed on different bottom electrodes as the nature of the electrode was found to be crucial for the performance.

## 1. Introduction

Nowadays, printed electronics have become an important research area with many interesting applications, such as displays, sensors or radio-frequency identification (RFID) tags. The different printing technologies are low-cost methods and can be used on flexible substrates, which makes them very attractive [1].

One basic part in printed electronics is the printing of capacitors, which are the most important energy-storage components in the electronic circuit technology [2,3,4]. Here, inkjet printing, as a solution-based maskless method, offers a fast and scalable way to deposit materials in a highly precise manner with low material wastage [5,6,7]. When printing on flexible substrates, ceramic/polymer composites can be used as a dielectric material. These composites have a good manufacturability and can have relatively high permittivities without the need for further thermal treatment [8,9,10,11,12]. For the application under ambient conditions, a temperature-independent permittivity is an important prerequisite. Ferroelectric ceramic powders that are in the nanometer range display a diffuseness of the permittivity with smaller grain size, which is advantageous for the printing of thin layers [13,14]. Furthermore, at low temperature, the permittivity stays almost consistent [15,16]. The higher the ceramic fraction, the higher the permittivity of the composite. The dependence of the permittivity on the ceramic ratio has already been examined by several authors and can be described by various models under appropriate assumptions [17,18,19,20]. Pure polymers have also been used as a dielectric in flexibly printed capacitors, but they only show low permittivities [21,22,23].

For example, Graddage et al. [21] used poly(vinylpyrrolidone) (PVP) as a dielectric material to print on PET (Table 1). Although PVP has a relatively low permittivity with a value of 3.9, very thin layers of 70 nm could be realized. Due to this, high capacitances could be achieved. The higher permittivities of 42–65 were obtained by Fu et al. [24] through the use of 2D Ca_2_NaNb_4_O_13_ perovskite nanosheets. Here, however, the substrate material was made out of glass, which makes the application in flexible electronics difficult. Through a composite system with BST and PMMA (poly(methyl methacrylate)) with a ratio of 66.6:33.3, Mikolajek et al. [9] was able to obtain a high permittivity of 42 with a layer thickness of 6 µm and with PET as a substrate. In our previous work [25], by reducing the thickness of the dielectric even further to 900 nm, an even higher capacitance could be obtained with a comparable permittivity of the composite material.

In order to use capacitors in electronic devices, it is essential to study the dielectric behavior closely. In addition to the capacitance, the loss factor, the leakage current and the dielectric strength are of interest for assessing the electrical component. The loss factor is a measure of the loss of electrical energy in the component, mostly by converting electrical energy into thermal energy. It is also known as the dissipation factor and is given by:

(1) $$DF=\tan\left(\delta \right)=\frac{1}{\omega RC}=\frac{\varepsilon^{\prime \prime }}{\varepsilon^{\prime }}$$

where *δ* is the loss angle, *ω* is the circular frequency, *R* is the forward slope resistance, *C* is the capacitance, *ε*″ is the dielectric absorption and *ε*′ is the relative permittivity [2]. 

The leakage current is a small current, which always flows in a real capacitor when a direct voltage is applied, although ideally the dielectric should behave like an isolator. This current is typically in the range of microamps and should not be too high, otherwise the energy consumption increases, and the component can heat up. Although the voltage is increased during the measurement of the leakage current, an irreversible breakdown of the capacitor can take place at one point. This point corresponds to the dielectric strength, expressed in voltage per thickness, which mainly depends on the dielectric used. It was found that the dielectric strength is independent of the layer thickness with the exception for very thin layers [26]. In addition, the electrodes used can play a major role in determining the dielectric strength or the leakage current and should always be considered [2].

Our previous work [25] successfully shows the applicability of the developed polymerizable ceramic ink system, which makes it possible to produce capacitors with very thin layers by suppressing the coffee stain effect. In the present study, a detailed examination of the dielectric behavior of the printed ceramic/polymer layers is carried out. Variations in the ink are intended to illustrate the potential of the composite used as a dielectric material.

The ink consists of surface-modified Ba_0.6_Sr_0.4_TiO_3_ (BST) particles, a polymeric crosslinking agent and a thermal radical initiator. The ratio of BST to polymer is varied, which leads to different permittivities of the printed composite layers. The printed layer thicknesses are varied as well, in order to check the reliability of the determined permittivity of the material. Finally, instead of a printed silver bottom electrode, a sputtered gold electrode is used to manufacture capacitors, in particular, to analyze the associated changes in leakage current and dielectric strength. These two quantities are crucial for evaluating the capacitors as electrical components.

## 2. Materials and Methods

Poly(ethylene glycol) diacrylate (PEG-DA; M_n_: 700) and 3-(Trimethoxysilyl)propyl methacrylate (TMSPMA) from Sigma Aldrich (St. Louis, MO, USA) were used as polymers, DOLACOL D1001 (Zschimmer & Schwarz, Lahnstein, Germany) as a dispersant and Dimethyl 2,2′-azobis(2-methylpropionate) (Wako Chemicals, Neuss, Germany) as thermal radical initiator.

*BST synthesis:* Through a sol–gel process, Ba_0.6_Sr_0.4_TiO_3_ powder was synthesized by dissolving barium acetate (0.422 mol) and strontium acetate hemihydrate (0.281 mol) in acetic acid (30.000 mol) under a nitrogen atmosphere. When stirring the solution, titanium isopropoxide (0.703 mol) was added and the obtained sol was subsequently diluted with water (181.800 mol). Afterwards, the sol was filtered (PTFE, 1 µm) and spray dried (MM-HT-ex laboratory spray dryer, Niro, Søborg, Denmark). Calcination of the precursor was performed in a tube furnace (CTF1600; Heraeus, Hanau, Germany) at 1100 °C for 2 h under purified dried air. Then, the calcined BST powder was milled in isopropanol (IPA) (40 wt%) with a laboratory stirred media mill (MiniCer, Netzsch, Selb, Germany) using 200 µm ZrO_2_ milling beads until the particle size was <200 nm. The solvent was evaporated at 60 °C under reduced pressure, and the powder was dried at 80 °C under vacuum.

*Surface modification:* In accordance with the synthesis of Xie et al. [27], the modification was performed by hydroxylating the BST powder (0.235 mol) with H_2_O_2_ (2.449 mol; 30 wt%). Firstly, the dispersion was sonicated for 30 min in a 1 L 3-neck round bottom flask and then heated up to 60 °C and stirred overnight. Then, the dispersion was centrifuged, and the obtained BST-OH powder was washed two times with water (3.333 mol) and dried at 80 °C under vacuum.

For the silanization of BST-OH (0.188 mol), the powder was dispersed in dried toluene (3.022 mol) under argon atmosphere in a 500 mL 4-neck round-bottom flask. Subsequently, TMSPMA (0.016 mol) was added, and the mixture is sonicated. After 30 min, the dispersion was heated up to 100 °C and stirred overnight. The next day, centrifugation was carried out and the BST-Si powder was washed twice with toluene (0.755 mol) and dried at 80 °C under vacuum.

*Ink preparation:* In the stirred media mill, the BST-Si powder (0.141 mol) was milled in IPA (0.499 mol) to deagglomerate the modified powder (particle size distribution: 39–197 nm; median: 72 nm). A 10 vol% dispersion was obtained by determining the solid content of the final ceramic dispersion via mass loss and calculating the necessary amount of butyl diglycol (BDG). In order to perform a solvent exchange, BDG and the dispersant (1.5 vol%) were added and IPA was removed under reduced pressure (60 mbar) at 60 °C. However, a subsequent determination of the solid content showed that a small amount of IPA remained in the dispersion, which resulted in an 8.5 vol% dispersion of BST-Si. For the preparation of the composite ink, PEG-DA was dissolved in IPA (20 vol%) and the polymer solution was mixed with the BST-Si dispersion in varying volume ratios of BST-Si to PEG-DA (Table 2). All dispersions were diluted with BDG, resulting in an overall solid content of each 10 vol%. Thus, the amounts of IPA and BDG vary for different volume ratios. However, this does not have an influence on the coffee stain effect with the exception of the 65 vol% BST-Si ink, which will be discussed below. Finally, the initiator was added directly before printing (1 mg/mL). For a ratio of 50:50, the ink shows a viscosity of 7.8 mPa*s and a surface tension of 26.9 mN/m. The viscosity was determined using a rotational rheometer (MCR 300; Anton Paar, Graz, Austria) with a cone–plate measurement geometry (d_cone_ = 50 mm, αcone = 2°) at a share rate of 1000 s^−1^ and 20 °C. The surface tension was measured using a force tensiometer (K100, Krüss, Hamburg, Germany).

*Inkjet Printing:* A single nozzle piezoelectric drop-on-demand inkjet printer (Autodrop Professional; Microdrop, Norderstedt, Germany) with a 70 µm nozzle was used. The piezo element was controlled via a rectangular signal with a negative voltage amplitude, and a negative gauge pressure of −10 mbar was applied on the ink vessel. For a stable droplet formation, a driving voltage of the piezo actuator of U_Head_ = 90 V and a pulse length of t_Head_ = 32 µs at an ejection frequency of 500 Hz were chosen. The printhead was heated to 25 °C, while the temperature of the substrate table was set to 70 °C. The composite areas of 5 × 5 mm^2^ were printed on untreated PET substrates (Melinex ST506, d = 175 μm, DuPont) using different drop spacing between 70–90 µm. The electrodes (2 × 8 mm^2^) were printed with a silver ink (Silverjet DGP-40LT-15C; Aldrich) using a 100 µm nozzle at 80 °C with a drop spacing of 130 µm for the bottom electrode and 100 µm for the top electrode. The effective area of the capacitors was measured to be 4 mm^2^, respectively.

*Sputtering:* The 2 × 8 mm^2^ bottom electrode was deposited as a 100 nm thick gold film by sputtering (Sputtering system Z 400, Leybold, Köln, Germany). To guarantee the adherence of the gold layer, a thin titan layer (10 nm) was sputtered first. The pressure was set to 5 × 10^−6^ mbar.

*Characterization:* SEM: Cross sections were prepared with an ion beam slope cutter (Leica EM TIC 3X, Leica Microsystems, Wetzlar, Germany) and analyzed via SEM (Supra 55, Carl Zeiss, Oberkochen, Germany) using an AsB detector or in-lens detector.

EDX: The elemental composition of a sample was analyzed by means of energy-dispersive X-ray spectroscopy using an Ultim^®^ Extreme (Oxford Instruments, Tubney Woods, UK). The device was integrated in the SEM, so that the images could be transmitted and analyzed accordingly. A resolution of ≤10 nm at 4 kV was used for the elemental analysis. In case of silver, only the L-line was interpreted.

Packing density: The packing density was analyzed using image analysis with ImageJ. The ceramic content was determined with an SEM image taken with the AsB detector, while the pores were determined with the in-lens detector. The polymer content was then calculated to achieve 100%.

Capacitance: The dielectric properties of the printed capacitors were analyzed via an impedance analyzer (E4980AL, Keysight Technologies GmbH, Böblingen, Germany) with a voltage amplitude of 1 V at a frequency of 200 kHz. This frequency was chosen in accordance with previous works [9] in order to ensure the comparability of the results. 

Leakage current and dielectric strength: The leakage current of printed capacitors was determined with a Precision Source/Measure Unit B2911A (Keysight Technologies GmbH, Böblingen, Germany). For this purpose, the resistance and the current were measured with an applied DC voltage. The applied voltage is initially 2 V and was increased in 0.5 V steps until the respective capacitor broke down, which was defined as showing a leakage current >1 mA. The corresponding electric field was registered as the dielectric strength of the capacitor.

## 3. Results and Discussion

The phase composition of BST was confirmed via X-ray diffraction (XRD), and the subsequent surface modification of the powder was verified by X-ray photoelectron spectroscopy (XPS) and thermogravimetric analysis [25]. The print-ready ink consisted of 5 vol% BST-Si, 5 vol% PEG-DA, an azo-initiator, IPA and BDG and was examined in regard to the polymerizability and the drying behavior. It was shown that the successful surface modification led to a fast increase in viscosity directly after the ink drop comes into contact with the heated substrate. Therefore, this leads to very homogenous layers, even when printing thin layers. The conceptualization of this work is based on the approach of Friederich et al. [28] and details concerning the production and the behavior of the ink can be found in our previous work [25]. 

**Figure 1:**
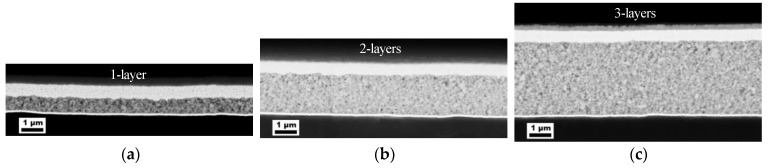
Cross sectional SEM images of different printed capacitors to determine the layer thickness of the dielectric. Layers were printed on a sputtered gold electrode with a drop distance of *p* = 90 µm with (**a**) one, (**b**) two or (**c**) three layers. An Ag electrode is printed on top.

First, in order to prove the reliability of the determined permittivity for the developed composite material, capacitors with different layer thicknesses were produced and analyzed by impedance spectroscopy. A sputtered gold layer was used as the bottom electrode, which has the advantage that they are uniform in comparison to the printed electrodes. This ensures that the composite layer is also of uniform thickness and that the calculated permittivity is correspondingly accurate. A detailed study of the influence of the bottom electrode on the performance of a capacitor is given later. The capacitance and the loss factor were measured directly, and the permittivity was calculated with the known effective area (2 × 2 mm^2^) and the layer thickness, which was determined via the cross sectional SEM images of ion-etched samples shown in Figure 1.

As can be seen in Table 3, the determined permittivities of the printed capacitors with different layer thicknesses match very well. Each layer was printed with a printing drop distance of 90 µm, resulting in the listed layer thicknesses. The permittivities lie between 40 and 44 (@ 200 kHz) with measured capacitances of about 450–2000 pF, so that a good reliability of the results can be assumed. The loss factors are between 0.10 and 0.15 and are similar for all layer thicknesses. However, previous studies showed that there can be a thickness dependence of the dielectric loss [29,30].

To demonstrate the flexibility of the printed composite ink and to find a possible maximum of the permittivity, the ceramic content in the ink was varied. For this purpose, five different inks with a total solid content of 10 vol% were compared. The volume ratios of ceramic to polymer compound were 20:80, 35:65, 50:50, 65:35 and 80:20, respectively. The printing drop distance was set to 70 µm for each ink and the printed Ag layers were used as bottom electrodes. The thickness and the microstructure were analyzed by cross sectional SEM images. To determine the porosity of the various composite materials, images were taken with an in-lens detector. Almost no material contrast could be seen in these images, but the pores could be identified very well and were analyzed using image analysis.

**Figure 2:**
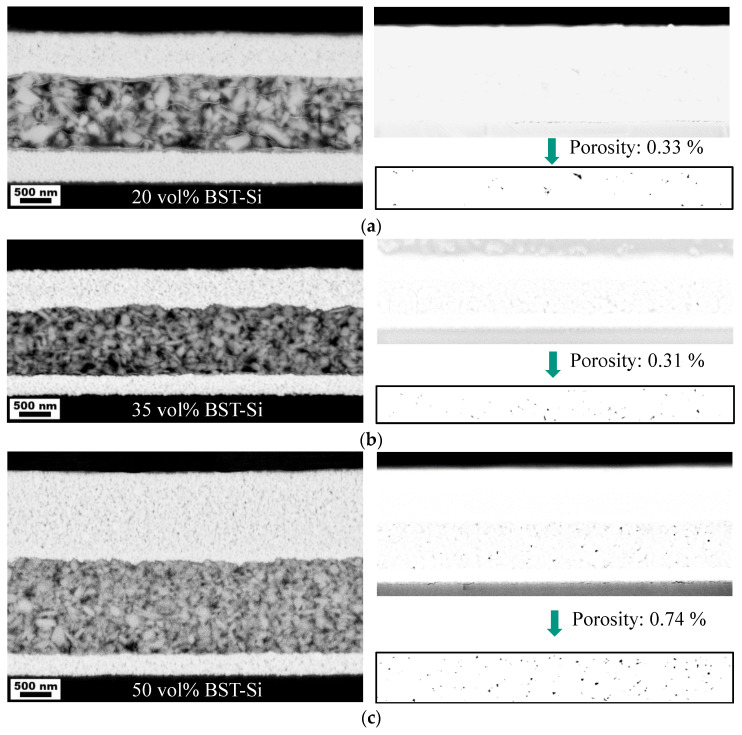

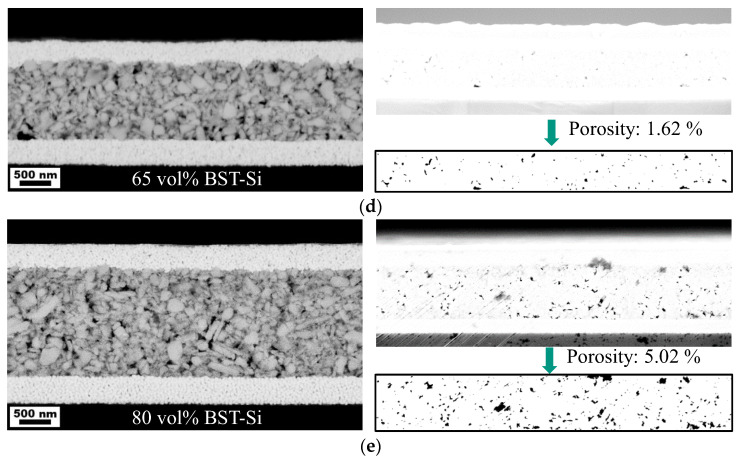
Cross sectional SEM images (AsB detector, left) and pore analysis (in-lens detector, right) of printed capacitors with increasing ceramic content of (**a**) 20 vol%, (**b**) 35 vol%, (**c**) 50 vol%, (**d**) 65 vol% and (**e**) 80 vol% BST-Si. All electrodes were printed with silver.

As expected, the amount of pores increases with increasing ceramic content. Due to their high packing density, polymers can form very thin layers during printing [31]. Therefore, with a low ceramic content, there should be very few to no pores. In addition, with higher ceramic content, the polymer can fill in the possible pores, which also leads to high packing densities [12,32]. In order to achieve high powder packing densities by random packing, it is advantageous to have particles with an aspect ratio smaller than one or even ellipsoidal particles [33]. The shape of the used BST-Si particles could, therefore, be helpful, as can be seen in Figure 2 (left). A broad particle size distribution can also be advantageous. A clear effect can be seen with a standard deviation of the particle radius of at least 15% [34]. A value of 15.3% was calculated for the given ink (median: 72 nm; standard deviation: 11 nm), so that an effect can be expected.

**Figure A1:**
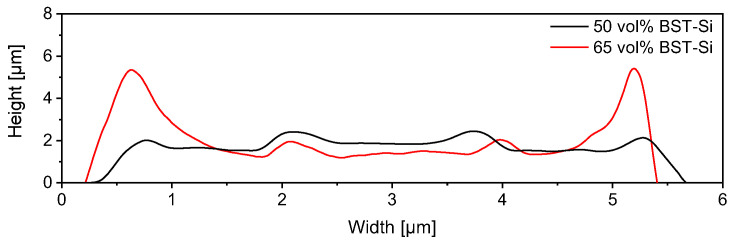
Depiction of the height profiles of the printed dielectric layers. For the layer with 50 vol% BST-Si, only a small elevation on the edge can be observed. In comparison, in the layer with 65 vol% BST-Si, a stronger elevation can be observed, which leads to a thinner layer in the center. However, it should be noted that the height is displayed in the µm range while the width is in the mm range.

The porosity for 20 and 35 vol% BST-Si is almost the same (~0.3%) and then increases steadily up to 5.0% at 80 vol% BST-Si (Figure 2, right). The resulting layers become thicker with increasing porosity, with the exception of the layer with 65 vol% BST-Si. Here, the layer thickness is the same as for the two layers with only 0.3% porosity, which is why these capacitors have the highest capacitance with the second-highest permittivity. This is due to the increased thickness on the edges of the printed area with 65 vol% BST-Si, which leads to a thinner layer in the center (Figure A1). Apart from the amount of pores, the ceramic fraction was also analyzed by means of image analysis using the SEM images with the AsB detector. The polymer fraction was then calculated to give a volume of 100%, so that the ceramic fraction could be interpreted as the packing density. The determined fractions and the actual ceramic content are shown in Table 4. The actual value of the ceramic content only relates to the ratio of ceramic to polymer, without considering any possible pore content. Thus, the actual value is comparable with the target value. All contents show only minor differences to the target values, except in the case of the 20% BST-Si sample. There, the ceramic content is overestimated, because some light-colored polymer filaments cannot be distinguished from the ceramic particles during image analysis (see Figure 2, left).

**Figure 3:**
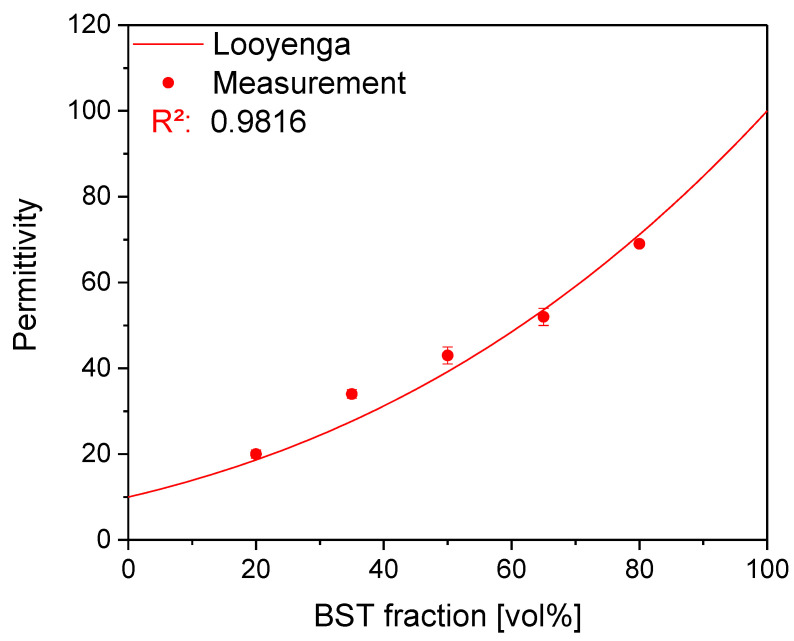
Simulation of the dependence of the permittivity on the ceramic ratio based on the model of Looyenga compared with the measured values.

As can be seen in Table 5, the permittivity increases with the increasing ceramic content as expected. As mentioned above, the capacitance does not increase in the same way because of the different layer thicknesses obtained. The thinnest layer is 1.2 µm thick and was achieved for 20, 35 and 65 vol% BST-Si, while the thickest layer is obtained for the highest ceramic content of 80 vol% BST-Si with a thickness of 1.8 µm. There are some models available to simulate the dependence of the permittivity on the ceramic to polymer/dielectric ratio, based on the permittivities of the pure components. The model of Looyenga [18] was found to be the best fit and is based on a 0–3 connectivity model [35], which assumes randomly dispersed ceramic particles in the polymer matrix. The coefficient of the determination R² of the experimental data for the Looyenga curve shown in Figure 3 is 0.9816.

The permittivity of PEG-DA was assumed to be 10 [36,37], while the permittivity of a non-sintered BST-layer was rated to be 100. The assumed permittivity for BST is very low compared to their sintered counterpart, which normally shows permittivities of around 1000 [38]. However, the permittivity of the bulk material cannot be obtained due to the presence of the pores. Therefore, the chosen value seems to be a good approximation for the used composite system, as was already shown by Mikolajek et al. [9]. They used a different composite system but with similar ceramic particles (same material and particle size) and could show that for an increasing particle size a higher permittivity has to be considered. In summary, the simulated curve in Figure 3 shows the range for the achievable permittivity with different BST-Si volume fractions and is in good agreement with the measured values displayed in Table 4.

**Figure 4:**
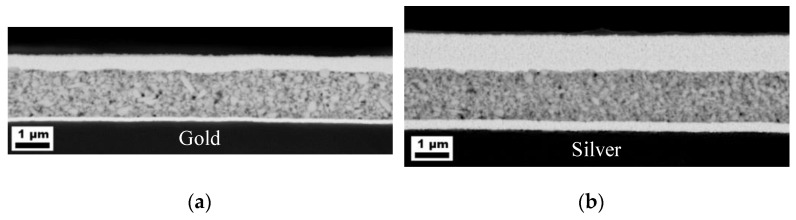
Cross sectional SEM images of printed capacitors with (**a**) gold and (**b**) silver bottom electrodes.

As already mentioned, the bottom electrode has a major influence on the dielectric behavior of a capacitor. Although the obtained permittivity must remain the same, the loss factor, leakage current and dielectric strength can differ [26,39,40]. In order to investigate this issue, capacitors with two different bottom electrodes were compared (Figure 4a) using the composite ink with 50 vol% BST-Si with a drop distance of 70 µm. The compared bottom electrodes consisted of sputtered gold and inkjet-printed silver, while the top electrode was always printed with the same silver ink. The printed composite layers on gold and silver look quite similar and only have a slightly different layer thickness, whereby the dielectric characteristics are nearly the same (Table 6). The difference in the layer thickness is due to a slight feathering of the ink on the gold electrode.

**Figure 5:**
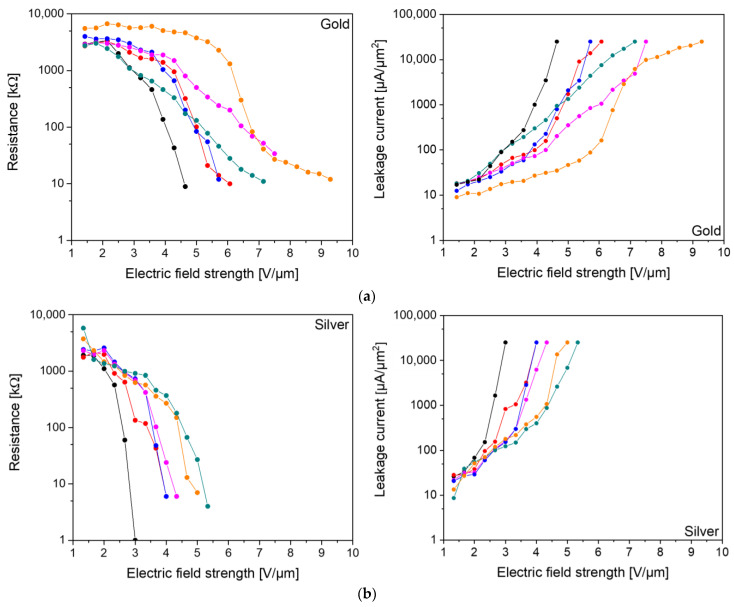
Resistances (left) and leakage currents (right) of printed capacitors with a (**a**) gold and (**b**) silver bottom electrode, respectively.

The measured resistances of the electrodes are 2 Ω for silver and 3 Ω for gold, and the leakage current and dielectric strength were measured for all types of capacitors. Even though the capacitors printed on gold and silver showed the same loss factors, the leakage current and the dielectric strength differed drastically. If the DC voltage was increased in 0.5 V steps, starting at 2 V, the best capacitor with a gold electrode broke down at 9.3 V/µm, while the best capacitor with a silver electrode already broke down at 5.3 V/µm. On average (six measurements each), the dielectric strength for gold was 6.7 V/µm and for silver was 4.3 V/µm (Figure 5). The starting values of the resistances and the leakage currents are similar for both types of electrodes, but the curve is steeper for silver. 

**Figure 6:**
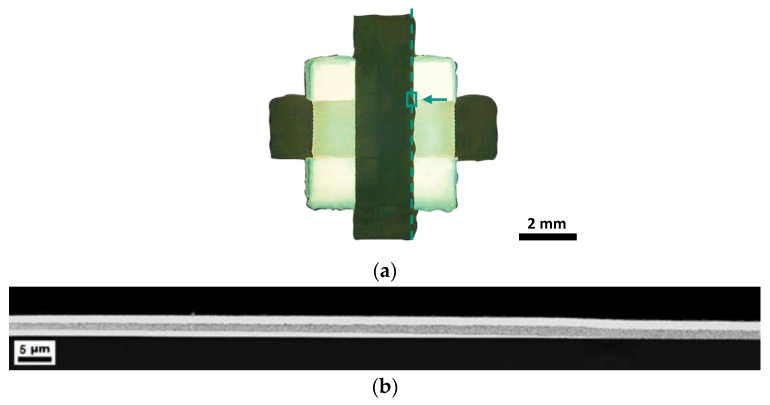
(**a**) Microscope image in which the dashed line shows the cut made to prepare the SEM image. The rectangle represents the area shown in the following cross-sectional SEM image and the arrow shows the viewing direction. (**b**) Overview of the boundary area of a silver capacitor, where the coffee stain effect can be seen at the silver bottom electrode.

The fast breakdown of the silver capacitors, when compared to capacitors with a gold electrode, can be explained when looking at the boundary area (Figure 6). The capacitor was cut along the dashed line (Figure 6a), and the following SEM image shows the marked area of the capacitor. Here, on the boundary, the thickness of the bottom electrode is 900 nm (Figure 6b), whereas the measured thickness in the middle area of the capacitor of is only 300 nm (compare with Figure 4). Due to the coffee stain effect of the bottom silver electrode, the thickness of the dielectric layer decreases from 1.5 µm in the middle to 1.0 µm at the boundary area. Therefore, the distance between the top and bottom electrodes is significantly smaller, and the breakdown occurs at a lower applied voltage. If the dielectric strength is related to the layer thickness of the boundary area of 1.0 µm, the average dielectric strength is 6.5 V/µm and is, therefore, almost identical to the dielectric strength shown for gold.

**Figure 7:**
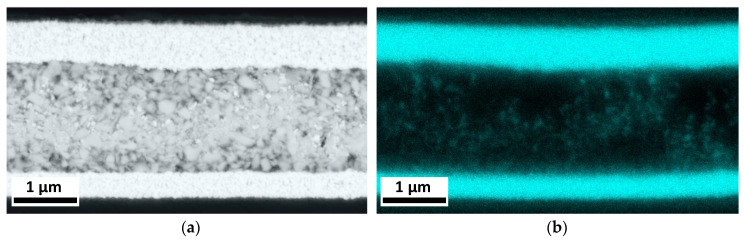
(**a**) Cross sectional SEM image of a silver capacitor after irreversible breakdown. (**b**) A silver veil formation can be seen with the help of EDX mapping.

In the end, an irreversible failure was found for all capacitors. To investigate a possible migration of silver and gold, the SEM images of samples after failure were analyzed in combination with the EDX mapping technique. First, the capacitor with a silver bottom electrode was examined, where the EDX clearly shows the presence of silver on the top and bottom electrode (Figure 7). Through the application of an external field, a distinctive veil formation of silver can be observed in the composite layer, which occurs due to the silver migration from both the top and bottom electrodes.

**Figure 8:**
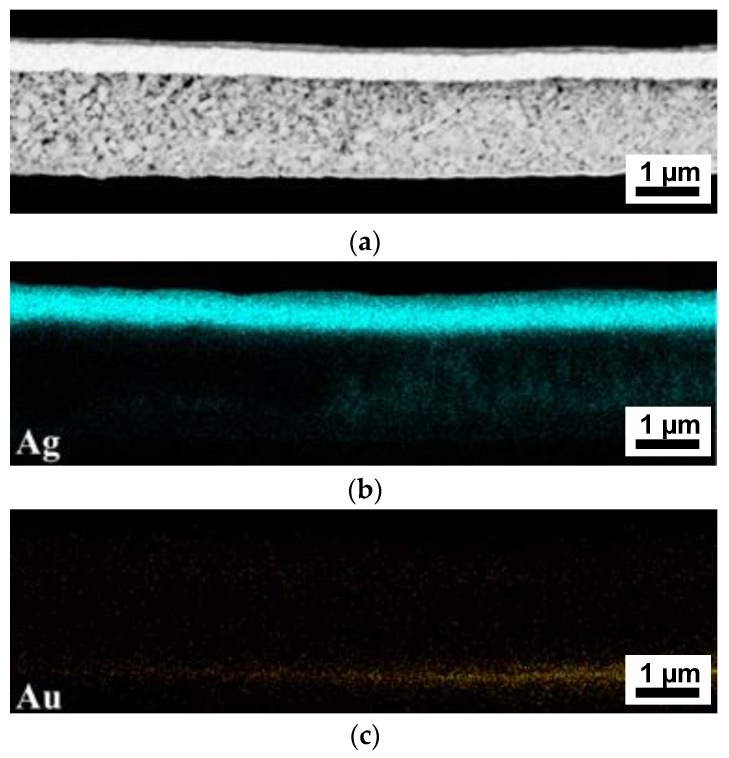
(**a**) Cross sectional SEM image of a gold capacitor after irreversible breakdown, with subsequent EDX-mapping, showing the (**b**) silver and (**c**) gold distribution.

Looking at the capacitor with gold as the bottom electrode, the same migration of silver particles can be seen from the top electrode, while no migration of gold occurred from the bottom electrode (Figure 8). In order to show that the migration of silver actually takes place during the breakdown, and only in the area where an electric field is applied, the edge region of the gold electrode is investigated. As can be seen, the silver particles only migrate into the composite layer if both the bottom and the top electrode are overlapping, thus enabling the formation of an electric field. The section of the composite layer without a gold electrode on the bottom shows no silver particles at all. Migration is generally driven by an electrochemical potential gradient that is generated when an electric field is applied. The magnitude of the electric field strength and the ion mobility are the main factors influencing migration [41]. Since the silver particles in the ink have a very small diameter (≤50 nm), they show a particularly high mobility in the electric field [42]. 

## 4. Conclusions

It was shown that the permittivity of the composite ink system consisting of surface-modified BST and PEG-DA remains stable for different layer thicknesses, as expected. The loss factors are also quite similar, ranging from 0.15 and 0.10. By varying the ratio of ceramic to polymer in the composite, it becomes clear that the permittivity increases with increasing ceramic content. As the simulation of the permittivity with the model of Looyenga [18] shows, a permittivity of 100 has to be considered for a printed, non-sintered BST layer with the given particle size, although the bulk material would have much higher values.

The achieved packing densities of the composite materials are very high, and even at a ceramic content of 80 vol% only 5.0% pores are present. This corresponds to an obtained packing density of 74.1% with the ceramic powder used. The broad particle size distribution (39–197 nm) and the shape of the particles could have been helpful in achieving this high value.

Finally, it has been shown that the choice of the bottom electrode is essential for the performance of the capacitor. The structure of this electrode influences the behavior of the leakage current and the dielectric strength. It is assumed that the coffee stain effect of the printed silver electrodes results in a lower dielectric strength compared to the sputtered, smooth gold electrodes. Thus, the printing of electrodes has to be optimized to get capacitors with better properties. Furthermore, it was shown that the breakdown of the capacitors is irreversible due to a migration of silver into the composite layer. Therefore, the development of electrodes should not be neglected, and alternative materials for the top electrode have to be found, e.g., carbon-based conductive inks, such as CNTs or copper-based electrodes. 

## Figures and Tables

**Table 1:** Comparison of the characteristics of inkjet-printed capacitors based on polymer and/or ceramic dielectrics.

Dielectric Material	Ratio (vol%) (cer./pol.)	Substrate	Thickness [µm]	Permittivity (Frequency)	Capacitance (pF/mm^2^)	Curing Temperature (°C)	Literature
PVP		PET	0.07	3.9 (100 kHz)	200	130 °C	[21]
BST + PMMA	66.6:33.3	PET	6	42 (1 kHz)	58	120	[9]
Ca_2_NaNb_4_O_13_ perovskite nanosheets		Glass	2	45–65 (1 kHz–1 MHz)	210	-	[24]
BST-Si + PMMA	50:50	PET	0.9	40 ± 1 (100 kHz)	500	120 °C	[25]

**Table 2:** Composite inks with different ceramic to polymer ratios, with an overall solid content of 10 vol% for each compound.

Ceramic:Polymer (Volume Ratio)	φ_Ceramic_ [vol%]	φ_Polymer_ [vol%]	φ_IPA_ [vol%]	φ_BDG_ [vol%]
20:80	2.0	8.0	35.6	54.4
35:65	3.5	6.5	32.3	57.7
50:50	5.0	5.0	28.9	61.1
65:35	6.5	3.5	25.5	64.5
80:20 *	8.0	2.0	17.8	72.2

* A 35 vol% PEG-DA solution was used here instead of 20 vol%.

**Table 3:** Capacitances, loss factors, permittivities and layer thicknesses of the different printed capacitors shown in Figure 1. All measurements were carried out at a frequency of 200 kHz.

Sample	Capacitance (pF)	Loss Factor	Permittivity	Layer Thickness (µm)	No. of Samples
1 layer	2000 ± 83	0.1257 ± 0.0250	40 ± 2	0.7	2
2 layers	857 ± 35	0.1132 ± 0.0041	44 ± 2	1.8	3
3 layers	451 ± 23	0.1161 ± 0.0140	42 ± 2	3.3	3

**Table 4:** Volume fractions of ceramic, pores and polymer determined via image analysis. The ceramic fraction from image analysis represents the packing density, while the actual ceramic content relates to the ratio of ceramic to polymer, without considering any pores.

Ceramic Content (vol%) (Target Value)	Ceramic ^1^ (vol%) (Packing Density)	Pores ^2^ (vol%)	Polymer ^3^ (vol%)	Ceramic Content ^4^ (vol%) (Actual Value)
20	23.87	0.33	75.80	23.95
35	36.12	0.31	63.57	36.23
50	50.03	0.74	49.23	50.40
65	62.20	1.62	36.18	63.22
80	74.12	5.02	20.86	78.04

^1^: Image analysis via SEM image with the AsB detector. ^2^: Image analysis via SEM image with the in-lense detector. ^3^: Calculated to give 100% with the ceramic and pore fraction. ^4^: Ceramic content, when neglecting pores.

**Table 5:** Capacitances, loss factors, permittivities and layer thicknesses of printed capacitors with different ceramic content, as shown in Figure 2. All measurements were carried out at a frequency of 200 kHz.

Ceramic Content (vol%)	Capacitance (pF)	Loss Factor	Permittivity	Layer Thickness (µm)	No. of Samples
20	596 ± 20	0.0928 ± 0.0135	20 ± 1	1.2	2
35	1004 ± 34	0.1066 ± 0.0004	34 ± 1	1.2	4
50	1010 ± 58	0.0895 ± 0.0025	43 ± 2	1.5	6
65	1527 ± 48	0.0805 ± 0.0013	52 ± 2	1.2	2
80	1364	0.1082	69	1.8	1

**Table 6:** Capacitances, loss factors, permittivities and layer thicknesses dependence of the electrode and the composite of printed capacitors with different bottom electrodes, as shown in Figure 4. All measurements were carried out at a frequency of 200 kHz.

Bottom Electrode	d_Electrode_ [nm]	Capacitance [pF]	Loss Factor	Permittivity	d_Composite_ [µm]	No. of Samples
Gold	100	1000 ± 72	0.0908 ± 0.0059	40 ± 3	1.4	6
Silver	300	1010 ± 58	0.0895 ± 0.0025	43 ± 2	1.5	6

## Data Availability

Not applicable.
